# Environmental protection subsidies, green technology innovation and environmental performance: Evidence from China’s heavy-polluting listed firms

**DOI:** 10.1371/journal.pone.0278629

**Published:** 2023-02-03

**Authors:** Chunyan Du, Qiang Zhang, Dekai Huang

**Affiliations:** 1 Financial Planning Department, China West Normal University, Nanchong, Sichuan, China; 2 Business School, Yunnan University of Finance and Economics, Kunming, Yunnan, China; 3 Yibin Vocational and Technical College, Yibin, Sichuan, China; East China University of Science and Technology, CHINA

## Abstract

The heavy-polluting industry is inexorably to responsible for the deterioration of the environment. Improving environmental performance is an unavoidable decision for heavy-polluting firms to ensure sustainable development under the policy framework of the carbon peak target. This study provides theoretical and empirical evidence for the effect of environmental protection subsidies on environmental performance. This study constructs basic and mediating effect models to measure how environmental protection subsidies affect environmental performance using panel data of China’s heavy-polluting listed firms from 2008 to 2019. This is an important outcome of industrial green transformation in environmental governance and provides a scientific basis for government departments to formulate environmental policies. The results of the empirical analysis show that environmental protection subsidies can improve the environmental performance of heavy-polluting listed firms. After receiving environmental protection subsidies, firms engaged in clean and green production through green technology innovation, thereby reducing external environmental pollution and improving their environmental performance. The mediating role of green technology innovation in the relationship between environmental protection subsidies and environmental performance is significant only in state-owned firms and firms in Eastern China. The research results may further guide the direction of green development of heavy-polluting industries, and thus promote harmonious development between the environment and the economy.

## Introduction

Green investment could be a good way to reduce environmental pollution by improving efficiency of energy conservation, expanding technological innovation capabilities and upgrading the industrial structure [1]. Although the government has increased investment in environmental governance in recent years from 338.73 billion yuan in 2007 to 1,063.89 billion yuan in 2020 and the expenditure on energy conservation and environmental protection has increased from 99.582 billion yuan in 2007 to 631.7 billion yuan in 2020, China still ranked 120th, with relatively low environmental performance in the "2020 Global Environmental Performance Index" released by Yale University and the World Economic Forum. This is a sign that China’s environmental situation still needs to be improved. To achieve the coordinated development of the economy and the environment, the "14th Five-Year Plan" Comprehensive Work Plan for Energy Conservation and Emission Reduction issued by the State Council stresses the goal that energy consumption per unit of GDP should drop by 13.5% and total emissions of chemical oxygen demand, ammonia nitrogen, nitrogen oxides, and volatile organic compounds should drop by more than 8%-10% in 2025, compared with them in 2020. "Made in China 2025" clearly proposes accelerating the green transformation of industries, and green technology innovation is key achieving the sustainable development of environmental protection. Since it is difficult to achieve rapid development by relying only on market forces, government intervention and regulation are necessary. Without sufficient environmental technology innovation and effective investment, heavy-polluting firms cannot engage in increasingly strict pollution control. They can adopt more effective measures to protect the environment in daily production activities with the help of the government’s reasonable guidance and incentives. This study discusses the impact of environmental protection subsidies on the environmental performance of heavy-polluting firms and uses the mediating effect model to test whether green technology innovation is a significant way for environmental protection subsidies to affect environmental performance. This discussion is of great theoretical and practical significance in improving the green innovation of heavy-polluting firms and promoting the guidance on environmental protection subsidies. This study further enhances the precision and applicability of environmental protection policies by serving as a data source for the government as it develops and implements environmental governance regulations.

The contributions of this study are as follows: (1) It provides novel insights that should inform policy-making related to subsidizing environmental protection. Existing research focuses more on environmental regulations or government subsidies in China more than environmental protection subsidies. However, this study takes environmental protection subsidies as the special funds to test whether environmental protection subsidies enhance the environmental performance of heavy-polluting firms. The findings of the study complement the literature on the heavy-polluting industries. (2) Few studies explore and test the mediating effect of green technology innovation on the relationship between environmental protection subsidies and the environmental performance of heavy-polluting firms. We contribute to the empirical literature by testing the ‘weak’ version of the Porter hypothesis. The research results are helpful for heavy-polluting firms to actively seek green development models in China and other similar emerging countries by utilizing environmental protection subsidies.

The remainder of this paper is arranged as follows. Section 2 reviews the literature on government subsidies, green innovation and environmental performance at home and abroad. Section 3 presents the research hypotheses, model specification, and variable settings. Section 4 discusses the results from the basic regression model, which is followed by a mediating effect analysis. Finally, the main conclusions, limitations, and policy implications of this study are highlighted in Section 5.

## Literature review

### Environmental performance factors

Clarifying corporate governance helps all of a firm’s stakeholders understand their respective roles, obligations, and interests. The creation and implementation of a firm’s environmental strategy are impacted by ownership concentration, institutional shareholding ratio, and board characteristics, which in turn impact the firm’s environmental performance. According to Earnhatt and Lizal (2006), ownership concentration has a favorable effect on environmental performance, and the more equity a state-owned firm has, the better its environmental performance [2]. However, Clarkson (2008) showed that ownership concentration had a detrimental impact on environmental performance using Singapore’s listed enterprises as a research sample [3]. Institutional shareholding might enhance a firm’s environmental performance, according to Li (2020) and Yu et al. (2017), and long-term institutional investors pay greater attention to environmental protection [4, 5]. According to Yousaf et al. (2022) [6], board capital has a favorable impact on firms’ green innovation. Boards and boards of director committees, according to Walls et al. (2012) and De Villiers et al. (2022), are concerned with environmental performance [7, 8]. Konadu et al. (2022) used data from the firms included on the Standards & Poor’s 500 index from 2002 to 2018 to examine how board diversity affected firms’ reductions in carbon emissions [9]. Their results support the theory that board characteristics influence environmental performance [10].

Shapiro and Walker (2018) noted that firms could be more active in environmental protection behavior only when the government implemented strict legislation [11]. Wang et al. (2021) suggested that only when the government exerts pressure on firms will they actively undertake environmental protection responsibilities, and the greater the pressure exerted, the better the environmental performance will be [12]. Environmental regulation is a restraining force by which the government adopts various policies to restrain individuals or organizations that have a negative impact on the environment to achieve the purpose of environmental protection [13]. Hu et al. (2022) and Tang et al. (2019) found that the environmental regulation made microenterprises promote environmental governance and green development [14, 15]. Xie et al. (2017) divided environmental regulation into command and incentive regulations, and suggested that the implementation of environmental protection regulation incentives could positively promote the green productivity of firms [16]. Shen and Zhou (2017) considered that China’s firms lack the motivation for environmental pollution problems, and external pressure from the government prompts firms to take short-term actions such as emission reduction [17]. Lin (2022) used the double-difference method to find that the environmental regulation of cleaner production can reduce the intensity of pollution emissions from firms based on the pollution discharge data of China’s industrial firms from 1998 to 2012 [18].

### Government subsidies and firms’ green innovation

Green innovation aims to reduce environmental pollution and save natural resources and energy [19]. Government subsidies are often recognized as a significant incentive tool for green innovation [20]. Liu et al. (2020) suggested that government subsidies have a positive role in promoting green process innovation [21]. Bai et al. (2019) examined whether government R&D subsidies increased the green innovation of China’s energy-intensive firms [22]. Ren et al. (2021) used an IV-2SLS model with data on China’s listed manufacturing firms from 2011 to 2015 to find that government subsidies significantly boosted firms’ environmental management innovation [23]. Chen et al. (2022) took the data of listed firms in the new energy industry from 2010 to 2020 as a sample and found that government subsidies can improve green innovation performance [24]. Some scholars hold opposing views. For instance, Sheng and Feng (2022) collected data on China’s heavy-polluting firms from 2010 to 2016 and showed that there is an inverted U-shaped relationship between government subsidies and corporate social responsibility [25]. Xia et al.(2022) found that government subsidies exhibited a U-shaped relationship with green innovation using panel data from new energy vehicle firms that acquired government subsidies from 2013 to 2018. Although government subsidies bring social benefits, they actually fail to fulfill their value in China’s firms [26, 27] due to the political connections [28], and the crowding-out and substitution effect [29]. Fan and Chu (2019) used 2012–2016 data on listed firms in China’s A-share heavy-polluting industries and found that more government subsidies would hinder firms’ green innovation from the perspective of the resource curse theory [30]. Given that the different effects of government subsidies on green innovation may coexist, it is essential to examine this relationship.

### Green innovation and environmental performance

Singh et al. (2020) noted that a firm’s environmental performance was affected by its process of green innovation using data from 669 manufacturing firms in the United Arab Emirates [31]. Kraus et al. (2020) utilized data from 244 Malaysian large manufacturing firms and found that green innovation was an important channel through which both green intellectual capital and human resource management promote a better environment [32]. Rehman et al. (2021) established that green innovation was related to environmental performance based on a sample of 244 large manufacturing firms [33]. Yan and Zhang (2021) verified that green innovation and environmental management had positive effects on environmental performance using a stochastic frontier model for energy-intensive listed companies from 2011 to 2017 [34]. Xie et al. (2022) and Zameer et al. (2021) stated that green process innovation could be seen as an effective solution that benefits the environment [35, 36]. Luo (2020) found that there was a significant positive correlation between green technology innovation and environmental performance [37]. Liang et al.(2022) examined the positive effect of technology innovation on the environmental performance of energy firms based on the data of 136 energy firms in China from 2009 to 2019 [38].

Prior research has primarily studied the influencing factors of environmental performance, the relationship between government subsidies and firms’ green innovation or the correlation between green innovation and environmental performance, and some results have yet to reach consensus. Although a few studies have focused on the relationship between government subsidies and environmental performance, they have failed to distinguish between government subsidies and environmental protection subsidies, and the mediating role of green technology innovation between them has not been confirmed. Environmental protection subsidies aim to protect the environment and control pollution. Discussing their impact on environmental performance has practical significance for exploring the effectiveness of implementing China’s environmental protection subsidy policy in the heavy-polluting field.

## Research design

### Research hypotheses

#### Environmental protection subsidies and environmental performance

Environmental pollution issues are mostly produced by heavy-polluting firms, which exhibit the traits of high investment, high energy use, and high pollution. In addition, the majority of heavy-polluting firms require many resources in their production and operation, which harms the environment’s ecology. In turn, stakeholders pay closer attention to the environmental performance of heavy-polluting firms due to their higher standards of environmental responsibility. The government provides society with important public products and services, and supervises the behaviors of firms through incentives and punishments. The Ministry of Environmental Protection of China pointed out in the "Key Points of Pollution Prevention and Control in 2011" that the environmental information disclosure system of listed firms needed to be improved and that environmental protection supervisions and penalties should be strengthened. The new institutionalism theory suggests that there are three "institutional pressures"(regulatory pressure, normative pressure and imitation pressure) that drive firms to undertake environmental protection responsibilities. Therefore, environmental regulation can significantly encourage firms to improve their environmental performance. However, through many empirical studies have revealed that command environmental regulation has not achieved the expected result [39, 40]. To implement environmental protection, firms need to pay additional costs. As "rational economic persons", firms would like to give up their environmental protection responsibilities in pursuit of maximizing economic benefits. Government subsidies include environmental protection subsidies, R&D subsidies, and talent subsidies [41]. Environmental protection subsidies, as special funds (such as energy-saving subsidies, energy-saving renovation bonuses, and environmental transformation subsidies), can reduce the operational risks of firms and improve their confidence in environmental activities. The environmental protection subsidies issued by the government send a signal of "environmental protection demand" to firms, and firms improve their environmental performance in response. Environmental protection subsidies belong to incentive environmental regulation, which is more beneficial for transmitting environmental information and helps firms obtain low-cost and differentiated competitive advantages and improve their economic performance [42, 43]. Incentive environmental regulation not only endows firms with opportunities to implement dynamic strategies, guiding them to optimize resource allocation, but also provides firms with greater incentives for energy conservation and emission reduction [44].

H1: There is a positive correlation between environmental protection subsidies and the environmental performance of heavy-polluting firms.

#### The mediating effect of green technology innovation

Green innovation refers to the innovative activities through which firms develop new technology or improve existing technological processes to achieve the sustainable development goal of win-win economic and environmental benefits [45]. According to the theory of government intervention, the government needs to intervene in the green innovation activities of “market failure” and “system failure”, which means that green technology innovation cannot simply rely on the financial support of the firm itself but also depends on the government subsidies and policy support. Environmental protection subsidies are the compensation and incentive for firms’ environmental protection actions. They are conducive to the sales and promotion of green products, and reduce the pressure of insufficient R&D investment. When firms receive government subsidies, their motivation for green innovation increases [46]. Environmental protection subsidies can make up for the shortage of funds faced by firms and stimulate their enthusiasm for green innovation. As a strategic tool to deal with environmental challenges, green technology innovation has a positive impact on the competitive advantage and sustainable development performance of firms [47]. On the one hand, green technology innovation alleviates the constraints of nonrenewable energy by clean energy production technologies, such as environmentally friendly materials, alternative energy and resource recycling, which can improve the utilization efficiency of resources and energy and reduce the pollution [48]. On the other hand, the pollutants emitted by firms in production are reduced by the introduction of terminal pollution control technology. By doing so, firms meet the government’s requirements for energy conservation and emission reduction, and establish a good green image in society [49]. Based on cost-benefit theory, green technology innovation can reduce resource consumption and production costs [50].

The "Porter hypothesis" proposes that firms can obtain benefits through technological innovation to compensate for their costs. Through environmentally friendly R&D technologies, firms gain a sustainable competitive advantage and ultimately realize the improvement of production efficiency and economic benefits. The Porter hypothesis is divided into the weak Porter hypothesis and the strong Porter hypothesis. According to weak Porter hypothesis, after receiving environmental protection subsidies, heavy-polluting firms are more willing to invest in green technology innovation and R&D, explore new development models required by the circular economy, and provide more competitive products to improve environmental performance.

Externality theory emphasizes that an economic subject’s economic action impacts other economic subjects without incurring any costs or receiving any compensation. Firms that take the lead in developing green technology innovation in the market cannot reap all of the benefits; other competitors can absorb the innovation results, which is the positive externality of green technology innovation and leads to a reduction in firms’ willingness to innovate. Environmental protection subsidies are a type of government support mechanism that can reduce the fluctuation of innovation investment to make up for lost profits resulting from positive externalities from innovation. This eventually increases the enthusiasm for technological innovation [51] and lowers the risk and cost of innovation [52] to improve environmental performance.

H2: Environmental protection subsidies affect the environmental performance of heavy-polluting firms through green technology innovation.

### Model specification and variable setting

The following models are constructed:

Basic model:

$$EP_{it}=\alpha_{0}+\alpha_{1}\;\text{ln}\;SUB_{it}+\alpha_{2}control_{it}+\sum Industry+\sum Year+\varepsilon_{it}$$ (1)

Mediating model:

$$\text{ln}\;GI_{it}=\beta_{0}+\beta_{1}\;\text{ln}\;SUB_{it}+\beta_{2}control_{it}+\sum Industry+\sum Year+\varepsilon_{it}$$ (2)

$$EP_{it}=\gamma_{0}+\gamma_{1}\;\text{ln}\;SUB_{it}+\gamma_{2}\;\text{ln}\;GI_{it}+\gamma_{3}control_{it}+\sum Industry+\sum Year+\varepsilon_{it}$$ (3)

Here, EP_it_ refers to the environmental performance of the i_th_ firm in year t; SUB_it_ is the government environmental protection subsidies of the i_th_ firm in the t_th_ year; GI_it_ represents green technology innovation of the i_th_ firm in year t; and control_it_ denotes a vector consisting of a series of control variables involving Lev, Roa, Share, Size, Age and Ic. Industry and Year are dummy variables. It is necessary to consider each model coefficient and its significance, which aids in investigating the relationship between environmental protection subsidies and environmental performance.

The explained variable, environmental performance (EP), can be measured by the score of the environmental protection strategy [5]. We collected disclosed information on environmental protection strategies, including environmental protection concepts, goals, management systems, education and training, special activities, emergency mechanisms, honors or awards, and the implementation of the "three simultaneous" system. If all of the above information is disclosed in an annual report, the value will be 1; otherwise, it will be 0.

The explanatory variable, environmental protection subsidies (lnSUB), is a type of policy intervention that is used by government departments to persuade firms to increase their investment in environmental governance or create environmentally friendly production methods to increase the competitiveness of their products. It can be expressed by the logarithm of government subsidies related to environmental protection, including cash subsidies and tax rebates.

The mediating variable, green technology innovation (lnGI), is a means for firms to achieve economic and environmental benefit advantages by upgrading technology and enhancing manufacturing processes. For heavy-polluting listed firms, the logarithm of the number of green patents granted can more realistically reflect the firm’s green innovation capability.

The following control variables are considered:

1. Lev is the ratio of a firm’s total liabilities to total assets.
2. Roe is the ratio of net profit to shareholders’ equity.
3. Share is measured using the shareholding ratio of the firm’s largest shareholder.
4. lnSize, is measured by the logarithm of the firm’s total assets.
5. lnAge is stated by the logarithm of the years in which the firm is listed.
6. lnIc, can be indicated by the logarithm of the internal control index from the DIB internal control and risk management database.

The specific measures of each variable are shown in Table 1.

**Table 1:** Measurement of each variable.

Name	Symbol	Formulas/Explanation
Environmental performance	EP	The average score of environmental protection strategy
Environmental protection subsidies	lnSUB	Log(environmental protection subsidies from government)
Green technology innovation	lnGI	Log(1+ the number of green patents granted)
Asset-liability ratio	Lev	Total liability/total assets
Return on equity	Roe	Net profit/shareholders’ equity
Proportion of the largest shareholders	Share	Number of shares held by the largest shareholder/total number of shares of the firm
Scale of the firm	lnSize	Log(total assets)
Age of the firm listed	lnAge	Log(1+years of the firm listed)
Internal control	lnIc	Log(internal control index score)

### Sample selection and data description

We select 16 types of heavy-polluting industries stipulated in the "Guidelines for Environmental Information Disclosure of Listed Firms" published by the Ministry of Ecology and Environment and take these heavy-polluting listed firms engaged in the above industries from 2008 to 2019 as the research samples, excluding firms with ST, *ST and missing data. Green patent data come from the CNRDS database, and other data come from the CSMAR database. Descriptive statistics of variables and a multicollinearity analysis are conducted using STATA software.

A total of 3681 sample observations are shown in Table 2. EP has a minimum value of 0, a maximum value of 1, and an average value of 0.4025. Although environmental conservation and ecological balance have been emphasized in recent years, firms’ environmental performance still needs to be improved. The lowest value of lnSUB is 5.9022, the highest value is 20.5724, and the average value is 13.8650, indicating that the Chinese government supports environmental actions for heavy-polluting firms. The value of lnGI ranges between 0 and 4.382, indicating that there is significant variance in green technology innovation across listed firms in the heavy pollution industry, with the majority falling below the average. The maximum value of Lev is 0.9970 and the minimum value is 0.0108, indicating that there is a significant difference in the debt-paying ability of sample firms, although the capital structure of heavy-polluting listed firms is rather fair. The minimum and maximum values of Roe are -50.0819 and 0.8743, respectively, indicating that most firms are profitable. The greatest value of Share is 89.99, the smallest value is 2.197, and there is a considerable gap between the firms, but the first largest shareholder in most firms distributes reasonably. The fact that lnSize might have a maximum value of 26.8397 and a minimum value of 19.2406 demonstrates how significantly the size of the firms varies. The internal control and governance of listed Chinese heavy-polluting firms are not significantly different according to the mean value of lnIc, which is 6.4866 with a standard deviation of 0.1554. A total of 1972 state-owned heavy-polluting listed firms make up 53.57% of the examined samples, whereas 1709 nonstate-owned heavy-polluting listed firms make up 46.43%. The results show that the maximum variance inflation factor (VIF) is 2.17, reflecting no multicollinearity between the variables.

**Table 2:** Descriptive statistics of each variable.

Variable	Obs	Mean	Std. Dev.	Min	Max
EP	3,681	0.4025	0.2450	0.0000	1.0000
lnSUB	3,681	13.8650	2.0888	5.9022	20.5724
lnGI	3,681	0.0802	0.3475	0.0000	4.3820
Lev	3,681	0.4702	0.2022	0.0108	0.9970
Roe	3,681	0.0300	0.9688	-50.0819	0.8743
Share	3,681	36.6093	14.9704	2.1970	89.9900
lnSize	3,681	22.4767	1.2629	19.2406	26.8397
lnAge	3,681	2.3527	0.6656	0.6931	3.3322
lnIc	3,681	6.4866	0.1554	4.9718	6.8816

## Empirical results and discussion

### Basic model test and mediating effect analysis

Table 3 shows the regression results of the relationship among environmental protection subsidies, green technology innovation and environmental performance. As indicated, environmental protection subsidies have a positive incentive effect on the environmental performance of heavy-polluting listed firms, and the coefficient of environmental protection subsidies is 0.0104 (p<0.01) in Model (1). Environmental protection subsidies have a significant positive impact on green technology innovation at the 1% level in Model (2). This means that a 1% increase in environmental protection subsidies will result in an increase in green technology innovation of 0.0088%. The coefficients of environmental protection subsidies and green technology innovation in Model (3) are 0.0102 and 0.0293, respectively, demonstrating that environmental protection subsidies affect environmental performance via green technology innovation. Environmental protection subsidies can not only directly increase the amount of funds held by heavy-polluting listed firms to fulfill their environmental protection obligations but also encourage heavy-polluting listed firms to achieve sustainable development and clean production by stimulating green innovation and R&D and ultimately improve the environmental performance. The empirical results strongly agree with H1 and H2.

**Table 3:** Results of the basic regression model.

	Model (1)	Model (2)	Model (3)
	EP	lnGI	EP
lnSUB	0.0104***	0.0088***	0.0102***
	(0.0020)	(0.0033)	(0.0020)
lnGI			0.0293***
			(0.0103)
Lev	-0.1268***	-0.0588*	-0.1250***
	(0.0218)	(0.0351)	(0.0218)
Roe	0.0020	-0.0023	0.0020
	(0.0036)	(0.0059)	(0.0036)
Share	0.0004	-0.0007*	0.0004
	(0.0003)	(0.0004)	(0.0003)
lnSize	0.0678***	0.0589***	0.0661***
	(0.0040)	(0.0065)	(0.0041)
lnAge	0.0110*	-0.0378***	0.0121*
	(0.0062)	(0.0100)	(0.0062)
lnIc	0.0779***	0.0598	0.0761***
	(0.0241)	(0.0388)	(0.0241)
_cons	-1.9077***	-1.6231***	-1.8601***
	(0.1583)	(0.2549)	(0.1590)
Industry	Y	Y	Y
Year	Y	Y	Y
N	3681	3681	3681
adj. R2	0.2663	0.0641	0.2677

Standard errors in parentheses

* *p* < 0.1,

** *p* < 0.05,

*** *p* < 0.01

Lev has a negative effect on environmental performance and is significant at the 5% level of significance, showing that the higher the asset-liability ratio is, the poorer the debt paying ability of the firm. As a result of the firm focusing more on profitability than on environmental management and technological R&D, the environment will perform better as a result of this market economy’s rational behavior. The coefficients of Roe and Share are insignificantly positive. This could be a result of the fact that environmental performance in high-polluting companies with environmental responsibility was not considerably improved by profitability and ownership structure. Because of the positive correlation between firm size and environmental performance, a larger firm is better able to compete and absorb losses, has lower environmental management costs due to economies of scale, and has more advantages for improving the firm’s environmental performance overall. Internal control may enhance the disclosure of green information and guarantee that firms deliberately uphold their responsibilities to protect the environment. Internal control systems are required, and firms must closely adhere to all applicable laws, regulations, and policies to engage in environmental operations.

### Robustness test

A robustness test is performed to increase the credibility of the results. There are numerous methods for testing robustness, such as alternative variables, supplementary variables, subsample regression, model substitution, and changing the sample size. Environmental protection investment (EPI) is used to measure the funds invested in environmental governance and ecological balance maintenance. As operational performance, environmental protection investment (EPI) can replace the score of the environmental protection strategy [5] in the robustness test (Model (1–3)-1). The environmental protection investment can mainly be collected from the notes on "construction in progress" in the firm’s annual report. It relates to environmental management, environmental protection design and energy savings, sewage treatment, desulfurization treatment, centralized energy monitoring and resource protection. For the robustness test, we replace the number of green patents granted in the model with the number of green patent applications (Models (1–3)-2). The above result is still likely to be endogenous because causality may not be confirmed or important variables may be omitted. Thus, environmental protection subsidies are lagged two years and green innovation is lagged one year to obtain Model (1–3)-3. The relevant empirical results are shown in Table 4. The direction of each variable is consistent with Model (1–3), which indicates that there is no serious endogenous problem in the basic regression model. With an increase in environmental protection subsidies, heavy-polluting listed firms actively engage in green technology innovation, which is favorable for environmental performance. Hence, the results of Model (1–3) are robust.

**Table 4:** Results of the robustness test.

	Model (1)-1	Model (2)-1	Model (3)-1	Model (1)-2	Model (2)-2	Model (3)-2	Model (1)-3	Model (2)-3	Model (3)-3
	lnEPI	lnGI	lnEPI	EP	lnGP	EP	EP	lnGI	EP
lnSUB	0.0695***	0.0088***	0.0666***	0.0104***	0.0100***	0.0102***			
	(0.0214)	(0.0033)	(0.0213)	(0.0020)	(0.0038)	(0.0020)			
lnGI			0.3492***						
			(0.0988)						
lnGP						0.0247***			
						(0.0088)			
L.lnSUB							0.0056**	0.0152***	
							(0.0026)	(0.0045)	
L2.lnSUB									0.0058*
									(0.0032)
L.lnGI									0.0254*
									(0.0148)
Lev	-0.4047*	-0.0588*	-0.3948*	-0.1268***	-0.1192***	-0.1238***	-0.1679***	-0.0696	-0.2372***
	(0.2271)	(0.0351)	(0.2266)	(0.0218)	(0.0410)	(0.0218)	(0.0282)	(0.0485)	(0.0361)
Roe	-0.0404	-0.0023	-0.0400	0.0020	-0.0020	0.0020	0.0015	-0.0014	0.0001
	(0.0306)	(0.0059)	(0.0305)	(0.0036)	(0.0068)	(0.0036)	(0.0043)	(0.0074)	(0.0402)
Share	0.0033	-0.0007*	0.0035	0.0004	-0.0010**	0.0004	0.0006*	-0.0010*	0.0006
	(0.0027)	(0.0004)	(0.0027)	(0.0003)	(0.0005)	(0.0003)	(0.0003)	(0.0006)	(0.0004)
lnSize	0.9006***	0.0589***	0.8776***	0.0678***	0.0735***	0.0660***	0.0751***	0.0644***	0.0788***
	(0.0417)	(0.0065)	(0.0421)	(0.0040)	(0.0076)	(0.0041)	(0.0052)	(0.0089)	(0.0063)
lnAge	-0.1365**	-0.0378***	-0.1232*	0.0110*	-0.0310***	0.0118*	0.0169*	-0.0610***	0.0196
	(0.0688)	(0.0100)	(0.0687)	(0.0062)	(0.0117)	(0.0062)	(0.0093)	(0.0160)	(0.0130)
lnIc	0.5587**	0.0598	0.5480**	0.0779***	0.1207***	0.0749***	0.0668*	0.0728	0.0340
	(0.2340)	(0.0388)	(0.2334)	(0.0241)	(0.0453)	(0.0241)	(0.0341)	(0.0587)	(0.0444)
_cons	-7.6603***	-1.6231***	-7.0765***	-1.9077***	-2.2687***	-1.8517***	-1.9325***	-1.8299***	-1.7313***
	(1.5613)	(0.2549)	(1.5662)	(0.1583)	(0.2975)	(0.1594)	(0.2178)	(0.3745)	(0.2877)
Industry	Y	Y	Y	Y	Y	Y	Y	Y	Y
Year	Y	Y	Y	Y	Y	Y	Y	Y	Y
N	2389	3681	2389	3681	3681	3681	2399	2399	1679
adj. R2	0.4110	0.0641	0.4138	0.2663	0.0641	0.2677	0.2594	0.0602	0.2600

The use of basic linear regression may lead to biased conclusions by overestimating the influence of subsidies on policy. Although the estimated coefficients might reveal a link between the environmental performance of firms and environmental protection subsidies, they are insufficient to prove that environmental protection subsidies are the cause of environmental performance. In this study, we employ the PSM method, which is based on estimating the propensity score, identifying a sample of firms in the control group that matches the sample firms in the treatment group, and then analyzing the impact of environmental protection subsidies by comparing the variations in environmental performance between the two groups after matching, which can successfully avoid the endogeneity caused by sample selection bias.

The equilibrium tests for the matched variables are shown in Table 5. As seen, the results of the t-test for all variables do not reject the original hypothesis that there are no systematic differences between the treatment and control groups, and the standardized deviations of most variables are significantly lower when comparing the results before matching. The standardized deviations of all variables after matching are less than 10%. Overall, matching can successfully remove the variations in the traits of the firms in the treatment and control groups.

**Table 5:** Equilibrium test for matching variables.

Variable	Unmatched	Mean			%reduct	t-test	
	Matched	Treated	Control	%bias	bias	t	p>|t|
Lev	U	0.4702	0.3971	36.2		16.69	0
	M	0.4700	0.469	0.5	98.6	0.23	0.822
Roe	U	0.0300	0.0558	-3.6		-1.79	0.074
	M	0.0436	0.0363	1	71.7	0.69	0.493
Share	U	36.609	35.513	7.3		3.34	0.001
	M	36.607	36.779	-1.1	84.3	-0.48	0.632
lnSize	U	22.477	21.976	39.3		18.07	0
	M	22.477	22.487	-0.8	98	-0.33	0.74
lnAge	U	2.3527	2.1591	27.7		12.69	0
	M	2.3526	2.3458	1	96.5	0.44	0.658
lnIc	U	6.4866	6.4829	2.3		1.05	0.293
	M	6.4867	6.4882	-0.9	58.7	-0.38	0.706

Using K neighbor matching (K = 1), radius matching, caliper K neighbor matching (K = 3), kernel matching, and local linear regression matching on the processing group and control group sample firms in Table 6, we explore the causal relationship between environmental protection subsidies and environmental performance. We then calculate the average processing effect of environmental protection subsidies on environmental performance. The average change in the environmental performance of the firms receiving environmental protection subsidies (the treatment group) and the average change in the environmental performance of the firms are represented by ATT (control group). The ATT coefficients in Table 6 are all positive, suggesting that firms’ environmental performance increases as a result of obtaining environmental protection subsidies.

**Table 6:** PSM estimation results of environmental protection subsidies and environmental performance.

	neighbor(K = 1)	radius	caliper K neighbor(K = 3)	kernel	local linear regression
Unmatched	0.0518***	0.0518***	0.0518***	0.0518***	0.0518***
	(0.0051)	(0.0051)	(0.0051)	(0.0051)	(0.0051)
ATT	0.0128*	0.0157***	0.017***	0.0181***	0.0143*
	(0.0076)	(0.0057)	(0.0063)	(0.0057)	(0.0076)

### Heterogeneity analysis

Table 7 details the mediating effect of green technology innovation in state-owned (Models (1–3)-4) and nonstate-owned (Models (1–3)-5) heavy-polluting listed firms. State-owned firms are the backbone of the national economy, and they bear more social responsibilities. In the implementation of national policies, state-owned firms should play the exemplary role of energy conservation and emission reduction. State-owned firms obtaining environmental protection subsidies would be more inclined to respond to the call of the local government, and they would increase environmental protection R&D innovation as well as energy conservation, emission reduction, pollution control, and environmental protection, conveying a determination to actively undertake social responsibility. It is more likely for state-owned firms to use environmental protection subsidies to improve production technology and environmental protection. In contrast, the social responsibility pressure of nonstate-owned firms is smaller. Driven by their own interests, nonstate-owned firms may allocate more resources to profit-making activities after obtaining environmental protection subsidies and cannot improve their environmental performance by conducting green innovation activities. Some nonstate-owned firms may fail to establish an effective supervision mechanism or internal control policy, which greatly weakens the positive relation between environmental protection subsidies and environmental performance.

**Table 7:** Meditating effect of green technology innovation in state-owned and nonstate-owned firms.

	Model (1)-4	Model (2)-4	Model (3)-4	Model (1)-5	Model (2)-5	Model (3)-5
	EP	lnGI	EP	EP	lnGI	EP
lnSUB	0.0079***	0.0104**	0.0075***	0.0131***	0.0068*	0.0133***
	(0.0027)	(0.0053)	(0.0027)	(0.0031)	(0.0036)	(0.0031)
lnGI			0.0433***			-0.0295
			(0.0116)			(0.0207)
Lev	-0.1612***	-0.1300**	-0.1556***	-0.0978***	-0.0027	-0.0979***
	(0.0293)	(0.0574)	(0.0293)	(0.0326)	(0.0385)	(0.0326)
Roe	0.0013	-0.0037	0.0015	0.0082	-0.0004	0.0082
	(0.0040)	(0.0079)	(0.0040)	(0.0075)	(0.0088)	(0.0075)
Share	0.0009***	-0.0011	0.0010***	-0.0006	-0.0003	-0.0006
	(0.0004)	(0.0007)	(0.0004)	(0.0004)	(0.0005)	(0.0004)
lnSize	0.0697***	0.0749***	0.0664***	0.0560***	0.0392***	0.0571***
	(0.0050)	(0.0098)	(0.0051)	(0.0068)	(0.0080)	(0.0068)
lnAge	0.0204*	-0.0714***	0.0235*	0.0072	-0.0295***	0.0063
	(0.0120)	(0.0235)	(0.0120)	(0.0087)	(0.0102)	(0.0087)
lnIc	0.0560**	0.0841	0.0524*	0.0986**	0.0186	0.0991**
	(0.0284)	(0.0556)	(0.0283)	(0.0432)	(0.0509)	(0.0431)
_cons	-1.8128***	-2.0473***	-1.7242***	-1.9019***	-1.0970***	-1.9343***
	(0.1874)	(0.3663)	(0.1883)	(0.2897)	(0.3417)	(0.2905)
Industry	Y	Y	Y	Y	Y	Y
Year	Y	Y	Y	Y	Y	Y
N	1972	1972	1972	1709	1709	1709
adj. R2	0.3560	0.0596	0.3602	0.1934	0.0981	0.1938

Table 8 demonstrates the meditating effect of green technology innovation in Eastern China (Models (1–3)-6), Central China (Models (1–3)-7)and Western China (Models (1–3)-8). As we can see, environmental protection subsidies have a positive effect on the environmental performance of heavy-polluting listed firms in Eastern China and the coefficient is 0.0114, which is significant at the 1% level. However, the coefficients of environmental protection subsidies in Central and Western China are not significant, indicating that environmental protection subsidies have not promoted the environmental performance of heavy-polluting listed firms. Some believe that environmental protection subsidies in Central and Western China cannot promote the environmental performance due to political connections [25]. Through Model (2)-6, it can be seen that the correlation coefficient of environmental protection subsidies in Eastern China is significant at the 5% level, indicating that heavy-polluting listed firms in Eastern China actively explore environmental protection technologies and strengthen green R&D after obtaining environmental protection subsidies and that heavy-polluting listed firms pursue green transformation and expanding technological innovation. In Model (3)-6, it is found that the coefficients of green technology innovation and environmental protection subsidies are significant at 0.0279 and 0.0111, indicating that green technology innovation plays a mediating role between environmental protection subsidies and environmental performance.

**Table 8:** Meditating effect of green technology innovation in different regions.

	Model (1)-6	Model (2)-6	Model (3)-6	Model (1)-7	Model (2)-7	Model (3)-7	Model (1)-8	Model (2)-8	Model (3)-8
	EP	lnGI	EP	EP	lnGI	EP	EP	lnGI	EP
lnSUB	0.0114***	0.0092***	0.0111***	-0.0118	0.0016	-0.0120	-0.0124	-0.0086	-0.0095
	(0.0021)	(0.0034)	(0.0021)	(0.0116)	(0.0148)	(0.0115)	(0.0141)	(0.0123)	(0.0137)
lnGI			0.0279***			0.1429			0.3336*
			(0.0104)			(0.1278)			(0.1774)
Lev	-0.1296***	-0.0595*	-0.1279***	-0.1286	0.0154	-0.1308	-0.0085	0.0730	-0.0329
	(0.0223)	(0.0360)	(0.0223)	(0.1497)	(0.1919)	(0.1492)	(0.2181)	(0.1908)	(0.2117)
Roe	0.0023	-0.0023	0.0024	-0.0585	0.0386	-0.0640	-0.0206	-0.0002	-0.0206
	(0.0037)	(0.0060)	(0.0037)	(0.0538)	(0.0690)	(0.0539)	(0.1728)	(0.1512)	(0.1675)
Share	0.0004	-0.0007	0.0004	0.0004	-0.0013	0.0006	-0.0019	-0.0020	-0.0013
	(0.0003)	(0.0004)	(0.0003)	(0.0017)	(0.0022)	(0.0017)	(0.0017)	(0.0015)	(0.0017)
lnSize	0.0679***	0.0594***	0.0663***	0.0174	0.0391	0.0118	0.0756**	0.0098	0.0723**
	(0.0041)	(0.0067)	(0.0042)	(0.0328)	(0.0420)	(0.0331)	(0.0326)	(0.0285)	(0.0316)
lnAge	0.0094	-0.0395***	0.0105	0.1325**	0.0253	0.1288**	0.0133	0.0063	0.0112
	(0.0064)	(0.0103)	(0.0064)	(0.0550)	(0.0705)	(0.0549)	(0.0527)	(0.0461)	(0.0511)
lnIc	0.0778***	0.0631	0.0761***	0.0499	-0.2725	0.0888	0.1428	0.1164	0.1039
	(0.0247)	(0.0399)	(0.0247)	(0.1891)	(0.2424)	(0.1916)	(0.2364)	(0.2068)	(0.2300)
_cons	-1.9108***	-1.6469***	-1.8649***	-0.4262	0.8455	-0.5471	-1.9231	-0.7921	-1.6588
	(0.1641)	(0.2653)	(0.1648)	(1.2283)	(1.5748)	(1.2289)	(1.5880)	(1.3892)	(1.5451)
Industry	Y	Y	Y	Y	Y	Y	Y	Y	Y
Year	Y	Y	Y	Y	Y	Y	Y	Y	Y
N	3564	3564	3564	58	58	58	59	59	59
adj. R2	0.2589	0.0548	0.2602	0.3057	-0.2878	0.3104	0.1020	-0.1268	0.1569

China’s economic development differs from region to region. Compared with Central and Western China, Eastern China has the advantages of a geographical environment, talent gathering and economic strength. In terms of technology innovation, the proportion of effective patents and innovation investment in Eastern China are generally better than that in Central and Western China. The fierce competition in the product market makes firms in Eastern China have a driving force for innovation and transformation and the strong awareness and motivation of technology innovation to achieve green development.

## Conclusions and policy implications

This study integrates the heavy-polluting list firm data of the CSMAR and CNRDS databases from 2008 to 2019 and measures the effect of environmental protection subsidies on environmental performance. The research conclusions are as follows. First,environmental protection subsidies promote environmental performance. The more government support a heavy-polluting firm receives, the more willing the firm is to actively undertake environmental protection responsibilities. This is mainly because environmental protection subsidies have a certain policy-oriented guidance. These subsidies can meet the capital needs of firms and share part of the costs of firms’ environmental protection responsibilities. Second, environmental protection subsidies indirectly affect the environmental performance of heavy-polluting listed firms through green technology innovation. That is, environmental protection subsidies can not only directly increase the capital holdings of firms for environmental responsibility but also achieve clean production by strengthening green technology innovation, thereby reducing environment pollution and improving the environmental performance. The mediating effect of green technology innovation is especially shown in state-owned firms and firms in Eastern China.

This study has limitations that it should be addressed by future research. First, there is room for a follow-up study on the effect of environmental protection subsidies on environmental performance in post-COVID-19, which may have surprising results and findings. Second, an investigation of environmental subsidies from nonlisted firms could be adopted to further extend the sample. Third, the nonlinear relationship or moderating effect could be explored. All of these studies could create breakthroughs for suggestions in the next step.

The findings also have some policy implications. First, green development is critical for China’s economic transformation [53]. Environmental pollution governance and environmental protection are long-term projects that require firms to invest more. To reduce the cost of firms, the government should increase environmental protection subsidies to introduce a pollution treatment system and strengthen cleaner production R&D. Second, heavy-polluting firms should combine the use of environmental protection subsidies with environmental information disclosure and establish a feasible environmental information disclosure system, which can not only improve the transparency of environmental information but also help the government and the public supervise environmental protection subsidies. Moreover, heavy-polluting firms should be actively engaged in green technology innovation and transformation to achieve clean production and low energy consumption. They should also focus on establishing strong ties with universities and other environmental protection firms to form strategic alliances on green technology R&D, especially for nonstate-owned firms and heavy-polluting firms in Central and Western China.

## Supporting information

S1 Data Regression data. (XLSX)
